# Schwann song of neurogenic mesenchymal stem cells: Identifying an alternative neural progenitor in adipose and bone marrow

**DOI:** 10.4103/NRR.NRR-D-25-00376

**Published:** 2025-08-13

**Authors:** Rhian Stavely, Leah C. Ott

**Affiliations:** Department of Pediatric Surgery, Massachusetts General Hospital, Harvard Medical School, Boston, MA, USA

Multipotent stromal cells, otherwise known as mesenchymal stem cells (MSCs), have been widely studied for their regenerative potential across multiple tissues, including the nervous system (Caplan, 2017). Reports suggesting that MSCs can differentiate into neurons and glia spurred optimism towards their future therapeutic application in nervous system disorders. Despite extensive research, however, the precise cellular mechanisms underlying their neural differentiation potential are unclear (George et al., 2019). Nevertheless, MSCs have been utilized in hundreds of clinical trials for neural regeneration following stroke, traumatic brain injury, spinal cord injury, multiple sclerosis, amyotrophic lateral sclerosis, Alzheimer’s disease, Huntington’s disease, and Parkinson’s disease among others (Andrzejewska et al., 2021). Albeit, MSCs appear to exert therapeutic effects without explicit cell engraftment, informing the prevailing hypothesis that they act via a ‘hit and run’ mechanism to stimulate regeneration of host tissues via their secretome, exosomes, or other pathways, rather than integrating into the regenerated tissue themselves (Caplan, 2017).

It is widely accepted that MSCs originate from the mesoderm and are derived from a perivascular stromal population of pericytes or fibroblasts (Vodyanik et al., 2010; Caplan, 2017; Soliman et al., 2021). As neurons and glia arise from ectodermal lineages, neural differentiation from mesodermal-derived MSCs would require a fundamental shift in established developmental trajectories both embryonically and postnatally. Krabbe et al. (2005) posited an alternative theory that neural differentiation observed in MSC cultures might not be intrinsic to MSCs but rather attributed to a distinct, yet unidentified, progenitor population. Studies, including our own, have identified the presence of neural crest (NC)-derived cells which can be cultured from bone marrow (BM) and subcutaneous adipose tissue (SAT) postnatally (Isern et al., 2014; Stavely et al., 2022; Ott et al., 2023). Embryonic NC cells are a multipotent population, contributing to diverse cell types across multiple organ systems. Their differentiation profile varies based on their axial origin: cranial (cephalic) NC gives rise to craniofacial mesenchyme, cartilage, bone, cranial neurons, glia, and connective tissue. Cardiac NC contributes to melanocytes, neurons, cartilage, and connective tissue of the third, fourth, and sixth pharyngeal arches. Truncal NC yields melanocytes, dorsal root ganglia, sympathetic ganglia, chromaffin cells of the adrenal medulla, and Schwann cells. Finally, vagal and sacral NC produce enteric neurons, enteric glia, parasympathetic neurons, and Schwann cells.

Here, we summarize the evidence that NC-derived Schwann cells can be isolated from SAT and BM as a distinct entity to MSCs. Notably, these cells may persist in MSC cultures, which may underlie observations of neural differentiation *in vitro*. This raises the possibility that prior reports of MSC neurogenesis may reflect the presence of distinct neural progenitors, rather than MSCs, undergoing neurogenic differentiation themselves. Appreciation of this distinction could provide an opportunity to refine our understanding of neural progenitor biology within the SAT and BM to optimize protocols for cell-based therapies. By delineating the origins of progenitor populations in these tissues, we can better harness their therapeutic potential for applications in neural regeneration and repair.

**Schwann cell origin of neural stem cells within subcutaneous adipose tissue:** Given its abundance and accessibility, SAT represents a valuable source of autologous cells for regenerative therapies. Motivated by reports of neural transdifferentiation in MSCs, we sought to interrogate the origin of this cell population and its potential therapeutic utility for enteric nervous system (ENS) disorders. The ENS, affectionately referred to as the “second brain,” is a complex network of neurons and glia localized within the smooth muscle and submucosa of the gut wall that intrinsically regulates most gastrointestinal functions. In mammals, the majority of the ENS is formed by vagal NC cells during early development with later contributions by sacral NC cells. Given our prior experience culturing enteric neural stem/progenitor cells, we more recently showed that postnatal ENS progenitors in the gastrointestinal tract arise from enteric glia, which are themselves derived from the NC (Stavely et al., 2021; Mueller et al., 2024). While adipocytes of the trunk are not NC-derived (Billon et al., 2007), we speculated that the cell population with neural differentiation potential from SAT may also have an NC origin. To test this, we utilized the Wnt1::Cre lineage-tracing model to label NC-derived cells and identified large NC-derived nerve fiber bundles that penetrated and traversed the posterior SAT of these mice (Stavely et al., 2022). By generating mice that co-expressed this NC tracer along with the glial marker Plp1-EGFP, we found that most, if not all, NC-derived cells in SAT are Schwann cells. When NC and non-NC derived cells were cultured independently, non-NC derived cells underwent adipogenesis in induction assays, whereas not a single adipocyte was produced by the NC-derived population. This indicated that the NC-derived cells of SAT are not a significant source of MSCs, whereas it is possible that a substantial population of Schwann-like cells can be cultured from SAT.

We have also transplanted neurospheres, generated from purified NC-derived cells, into the gut, and shown successful engraftment, integration within the ENS, and differentiation into functional neurons of differing neurochemical coding (Stavely et al., 2022). Given their proliferative potential and multipotency, we designated this population as SAT-neural stem cells (SAT-NSCs). In further experiments, we optimized SAT-NSC isolation via counter filtration methods of nerve fiber enrichment and verified their presence in human tissues. Transcriptional profiling revealed SAT-NSCs express multiple neurotrophic factors, while transplantation studies confirmed their neural function as they partially reversed dysmotility in mouse models of gastroparesis and Hirschsprung disease.

**Figure 1:**
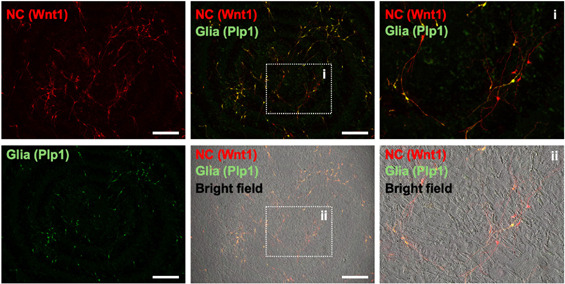
Presence of neural crest-derived glia (Schwann cells) in MSC cultures. Fluorescence of the neural crest tracer Wnt1::tdTomato (red), and glial/neural progenitor marker Plp1-EGFP (green), in cells cultured from the posterior SAT of mice. Cells were cultured in standard conditions for MSC isolation including plastic adherence in alpha-minimum essential medium supplemented with 16.5% fetal bovine serum, 1% GlutaMAX supplement, and 1% penicillin streptomycin. Images are taken in the second passage. Scale bars: 500 μm. Unpublished data. EGFP: Enhanced green fluorescent protein; MSC: mesenchymal stem cells; NC: neural crest; SAT: subcutaneous adipose tissue.

Notably, when generating cultures using conventional MSC isolation and expansion methods, we observe the persistence of these non-MSC, NC-derived cells that express the glial marker Plp1-EGFP, even after multiple passages (**Figure 1**). This raises the possibility that SAT-NSCs can persist in MSC cultures, leading to the presence of glia and neurons. Without diminishing the importance of previous contributions in this field, the assertion that MSCs from SAT undergo neural differentiation may warrant careful re-evaluation in light of this newly identified SAT-NSC population. These findings may have inadvertently affirmed the presence of a distinct neural progenitor population within MSC cultures as postulated by Krabbe et al. (2005), at least in the context of SAT.

**Adult neural stem cells derived from neural crest and Schwann cells in bone marrow:** The BM is another attractive tissue source for autologous cell therapies, and has been extensively studied as a reservoir of MSCs with both neurogenic potential and neurotrophic properties. To understand the developmental origins of MSCs and bone, Isern et al. (2014) utilized an NC tracing model to investigate possible NC contributions to this tissue. Their findings revealed a partial NC origin for osteoblasts, osteocytes, and chondrocytes in limb bones, suggesting the presence of an NC-derived MSC population. Within the BM, distinct NC-derived cell populations were identified, including Gfap^+^ Schwann cells, Nestin^+^ Pdgfra^-^ cells, and Nestin^+^ Pdgfra^+^ cells. It should be noted that Nestin is not a specific marker of neural stem/progenitor cells and is expressed by other cell types with regenerative capabilities, including pericytes and endothelial cells. The Nestin^+^ population localized to a perivascular niche, aligning with prevailing views on the anatomical origins of MSCs. The Nestin^+^ Pdgfra^-^ subset displayed high expression of Schwann cell precursor-like markers, including Sox10, Plp1, Erbb3, and Dhh, yet lacked expression of the mature Schwann cell marker Gfap. While Nestin^+^ Pdgfra^+^ cells were capable of adipogenesis but not gliogenesis, the Nestin^+^ Pdgfra^-^ population exhibited the inverse differentiation potential. These findings underscore two key insights: (1) though bone and cartilage primarily arise from the mesoderm during development, a subset of NC-derived Nestin^+^ Pdgfra^+^ MSCs contribute to these structures postnatally and (2) a distinct population of cells reminiscent of embryonic Schwann cell progenitors can be cultured from BM *in vitro*.

To specifically examine the BM vasculature, we employed a procedure to flush and isolate intact blood vessels from long bones, the presumptive niche for MSCs (Ott et al., 2023). We utilized the Plp1-EGFP glial reporter mouse model to visualize Schwann cells within this tissue and identified a population of Plp1-EGFP^+^ cells residing in the perivascular space. Specifically, these cells were found within autonomic nerve fibers, which travelled along and directly apposed the BM vasculature. Using the Wnt1::Cre mouse model, we determined that the NC predominantly contributed to this anatomical niche through these nerves with TUBB3^+^ axons and closely associated MPZ^+^ Schwann cells. When cultured under free-floating conditions, cells from these nerve fibers dramatically upregulated expression of NC markers *Ngfr* and *Nestin*. Furthermore, upon transplantation of neurospheres derived from these cultures into mouse gut *in vivo*, we observed that the transplanted cells survived, differentiated into TUBB3^+^ neurons, and integrated into the host enteric ganglia of the ENS.

Together, these findings support the presence of multiple progenitor populations within BM. Notably, a distinct glial population resides in the perivascular space, the niche thought to be occupied by MSCs. Given the findings of Isern et al. (2014), however, NC-derived cells with a Schwann-like profile lack MSC trilineage differentiation capacity, suggesting they represent a unique progenitor entity. Furthermore, the NC-derived population within BM appears to be the sole subset with neurogenic potential *in vitro*.

**A word on nomenclature and Schwann cell plasticity:** While MSCs have transitioned from “stem cell” to “stromal cell” nomenclature, we consider the term “neural stem cell” to be appropriate here for several reasons. The shift in MSC terminology arose from concerns that “stem cell” might imply direct differentiation into regenerating tissue, whereas MSCs primarily mediate repair through paracrine or other mechanisms besides engraftment (Caplan, 2017). In contrast, we demonstrated that SAT/BM-NSCs exhibit high engraftment rates and differentiate into appropriate cell types that contribute to the architecture of recipient tissue, distinguishing them from most MSC applications. By strict definition, “stem cell” implies the capacity for indefinite self-renewal, a property yet to be fully examined in SAT/BM-NSCs. Yet it remains widely accepted for hematopoietic stem cells (HSCs) despite their limited proliferative potential. Given these considerations, we believe the NSC terminology is justified and better reflects their engraftment capacity in the context of regenerative medicine.

Given that glia in the periphery are Schwann cells and SAT/BM-NSCs are derived from a glial population, it may be tempting to instead refer to them as Schwann cell precursors or Schwann cells. The term “Schwann cell precursor” has been specifically reserved, however, for a distinct population of NC cells present during embryonic and fetal development that give rise to Schwann cells among other derivatives (Solovieva and Bronner, 2021). Postnatally, Schwann cells have remarkable plasticity, particularly in response to injury or changes in their microenvironment. As neurons do not exist in BM or SAT, neuronal differentiation potential is not within their specific *in vivo* differentiation paradigm. However, our data indicates that SAT/BM-NSCs may represent a unique *in vitro* phenomenon resulting from the cell isolation process and exposure to subsequent culture conditions. For example, when nerve fiber bundles are isolated and cultured from SAT, the expression of *Nestin* is initially limited. Within 3 days, intense *Nestin* expression is apparent in the Schwann cells, which is far too rapid to be accounted for by the expansion of a small *Nestin*-expressing subset. Further, the induction of *Nestin* coincides with the loss of mature Schwann cell markers and increased expression of earlier NC and stem cell markers. Based on these observations, we proposed that “mature” Schwann cells themselves “dedifferentiate” into an NSC population, a process that may be normally biologically irrelevant *in vivo*, yet holds significant therapeutic potential.

**Figure 2:**
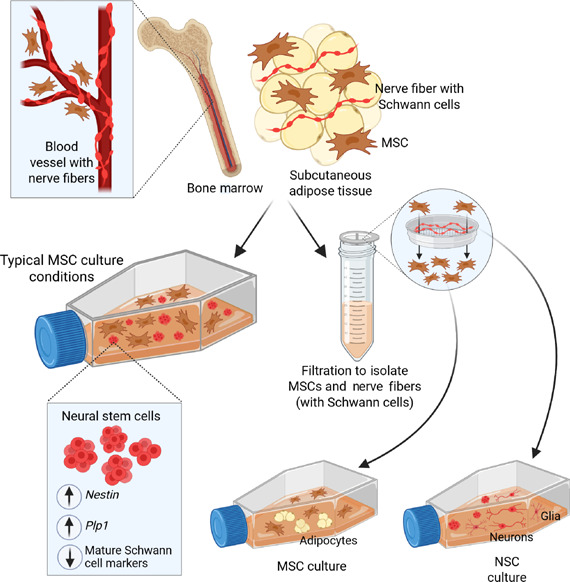
Summary of mesenchymal stromal cell and neural stem cell biology within the bone marrow and subcutaneous adipose tissue. Bone marrow contains both mesenchymal stromal cells (MSCs) and Schwann cells, the latter within nerve fiber bundles residing in the perivascular space directly apposed to blood vessels. Subcutaneous adipose tissue similarly contains MSCs and Schwann cells within resident nerve fiber bundles. Schwann cells may persist under typical MSC culture conditions, forming neural stem cells (NSCs) that upregulate *Nestin* and express *Plp1* (reflective of their Schwann cell origin) while downregulating mature Schwann cell markers. MSCs can undergo adipogenesis but not neuronal differentiation, while NSCs cannot differentiate into adipocytes but may give rise to neurons and glia. Created with BioRender.com.

**Reevaluating MSCs as neural progenitors and the Schwann cell alternative:** Both MSCs and NSCs from BM and SAT offer great promise clinically as autologous cell therapies. The findings discussed here, however, challenge the prevailing dogma that MSCs possess an intrinsic capacity for neurogenic and gliogenic differentiation. Instead, we propose that Schwann cells, present in both tissues, give rise to the neural phenotypes previously attributed to MSCs (**Figure 2**). This distinction is critical for clinical translation, as it suggests previously reported neural differentiation could have been misattributed to MSCs, rather than to NSCs originating from resident Schwann cells. As such, the presence of Schwann cells as a potential “contaminant” in MSC cultures should be carefully considered in past, present, and future studies. A clearer distinction between MSCs and NSCs is essential to refine cell therapies for neural regeneration and repair, ensuring the most relevant progenitor populations are optimized for therapies aimed at replacing or supplementing neural tissue with transplanted cells. Notwithstanding, much of the MSC literature has shifted toward cell-free approaches involving secreted factors and extracellular vesicles to promote endogenous tissue repair. NSCs could possess a secretome more relevant than MSCs for regeneration after nerve injury. For instance, human SAT-NSCs isolated from nerve fiber bundles exhibit significantly higher expression of neurotrophic factors such as GDNF and BDNF than cells from the stromal vascular fraction harboring MSCs (Stavely et al., 2022). Adipose-derived MSCs in particular have been an attractive source for nerve regeneration due to their accessibility and have shown promise in preclinical models of peripheral nerve regeneration (Qin et al., 2023), and a recent Phase I clinical trial for spinal cord injury, which showed the approach was well tolerated, with encouraging signs of sensory and motor recovery (Bydon et al., 2024). These findings validate MSC safety and feasibility, but they also establish a strong foundation for the next generation of cell-based therapies with potentially greater regenerative potency. Comparative studies between NSC and MSC could determine whether a shift in therapeutic focus is warranted, and potentially reveal the most effective strategies for neural regeneration.

*We would like to thank Dr Alan J. Burns (Massachusetts General Hospital, USA) for his thoughtful feedback on the manuscript.*
